# Morphine Exposure and Enhanced Depression-like Behaviour Confronting Chronic Stress in Adult Male Offspring Rat

**DOI:** 10.32598/bcn.9.10.155

**Published:** 2019-07-01

**Authors:** Anahita Torkaman-Boutorabi, Fereshteh Seifi, Ardeshir Akbarabadi, Heidar Toolee, Mitra-Sadat Sadat-Shirazi, Nasim Vousooghi, Mohammad-Reza Zarrindast

**Affiliations:** 1. Department of Neuroscience, School of Advanced Technologies in Medicine, Tehran University of Medical Sciences, Tehran, Iran.; 2. Research Center for Cognitive and Behavioral Sciences, Tehran University of Medical Sciences, Tehran, Iran.; 3. Department of Biology, Faculty of Biological Sciences, North Tehran Branch, Islamic Azad University, Tehran, Iran.; 4. Faculty of Veterinary Medicine, Garmsar Branch, Islamic Azad University, Garmsar, Iran.; 5. Iranian National Center for Addiction Studies (INCAS), Tehran University of Medical Sciences, Tehran, Iran.; 6. Department of Anatomy, School of Medicine, Tehran University of Medical Sciences, Tehran, Iran.; 7. Department of Pharmacology, School of Medicine, Tehran University of Medical Sciences, Tehran, Iran.

**Keywords:** Depression, Chronic stress, Corticosterone, Morphine, Offspring

## Abstract

**Introduction:**

Opioid addiction is an important concern in the World. Reports demonstrate that substance use disorder could influence genetic and environmental factors, and children of addicts have a higher rate of psychopathology. In this study, we investigated depression-like behavior among offspring of morphine-exposed rat parents.

**Methods:**

Adult male and female Wistar rats received morphine for 21 consecutive days and then let them were free of drugs for ten days. Offspring of these rats were divided into three distinct groups: maternal morphine-exposed, paternal morphine-exposed, and both maternal and paternal morphine-exposed. We used sucrose preference and Forced Swim Test (FST) to measure depression-like behavior. Also, we induced chronic mild stress using repeated corticosterone injection and evaluated depression-like behavior in offspring of morphine-exposed parents compared with offspring of healthy ones.

**Results:**

Results indicated that depression-like behaviors in the offspring of morphine-exposed rats were higher than those in the offspring of the control group in confronting with chronic mild stress. Additionally, mild chronic stress can produce an exaggerated effect on depression-like behavior in offspring of the morphine-exposed parent(s) compared with those of the control group.

**Conclusion:**

Our data support the previous hypothesis that the depression rate is higher in the children of addicts. We verified that even when mother or father was clean of opioid in the time of gestation, their children would be susceptible to depression. Dysregulation of hypothalamic-pituitary-adrenal axis and changing in neuronal features in the hippocampus increased depression-like behavior in the offspring of morphine-exposure parents.

## Highlights

- Depression in the offspring of morphine-exposed rats is higher than the offspring of the control group.
- Chronic stress produces an exaggerated effect on depression in offspring of morphine-exposed rats.
- Depression rate is higher in the children of addicts.

## Plain Language Summary

Addiction is a multifactorial disorder with genetic, neurobiological, and psychosocial aspects. Epigenetic changes are explained as an alteration in gene expression without altering in DNA sequences. Factors such as stress and substance abuse can cause epigenetic changes and produce psychiatric behaviors. Based on some evidence, 50% of hospitalized children for the psychiatric disorder had addicted parents. Alcohol-abusing parents increase the risk of mental health problems such as depression, suicidal attempts, antisocial behavior, and drug abuse in children. In this study, we investigated the effect of parental (maternal and or paternal) morphine exposure 10 days before gestation on the depression state of the offspring. We also evaluated chronic mild stress-induced depression in offspring by using chronic corticosterone injection as a model of mild chronic stress. We found that parental morphine exposure induced depression in offspring. Morphine can directly affect epigenetic germ cells, and this causes developmental changes in the embryo. The mechanisms which induce such trans-generational effect are unknown, and other research should be done to show the mechanisms of such effects.

## 1. Introduction

Addiction is a multifactorial disorder with genetic, neurobiological, and psychosocial correlates (Goodman, 2008; Hurd, 2006). Drug addiction is a severe, chronically relapsing neuro-psychiatric disorder. According to Koob and LeMoal definition, addiction is characterized by the compulsion to seek and take drugs, the loss of control over limiting the intake, and the occurrence of a negative emotional state reflecting a withdrawal syndrome when there is no access to the drug (Koob & Le Moal, 1997).

Depression is comorbid with addiction and using illegal drugs as self-medication for depression, but prolonged drug use can exacerbate the severity of depression and increase drug intake (Bruijnzeel, Repetto, & Gold, 2004; Crist et al., 2013). Stress is a major environmental factor which is usually seen in the etiology of mood disorders (Charney & Manji, 2004; Paykel, 2003). Stress strongly implicated in both precipitation of depressive episodes (Kendler, Thornton, & Gardner, 2000; Willner, Scheel-Kruger, & Belzung, 2013) and relapse in drug abuse (Shaham, Shalev, Lu, De Wit, & Stewart, 2003).

There is considerable overlap in the neurobiological substrates of depression and addiction (Bruijnzeel et al., 2004; Markou, Kosten, & Koob, 1998). Epigenetic changes are described as an alteration in gene expression without altering in DNA sequences, which include changes in DNA methylation, histone modifications, and micro-RNA. Factors such as stress and abusing drugs could induce epigenetic changes and affect neuropsychiatric behaviors (Bagot, Labonte, Pena, & Nestler, 2014; Nestler, 2014; Robison & Nestler, 2011).

Based on some evidence, 50% of hospitalized children for the psychiatric disorder had addicted parents. Alcohol-abusing parents increase the risk of mental health problems such as depression, suicidal attempts, antisocial behavior, and drug abuse in children (Balsa, Homer, & French, 2009; Christoffersen & Soothill, 2003).

Cortisol level increases during stress and in a depressed patient (Dinan, 1994; Gold, Goodwin, & Chrousos, 1988; Reus & Miner, 1985; Sachar et al., 1973). Johnson and colleague used repeated corticosterone injection as a chronic stressor for creating a rat model of depression following stressful events (Johnson, Fournier, & Kalynchuk, 2006). Previous investigations revealed that chronic stress would lead to anhedonia-like symptoms like reduced preference in sucrose (Wallace et al., 2009; Wook Koo et al., 2016). Our previous research established that parental morphine exposure before mating could affect anxiety-like behavior in the first generation but not in the second generation (submitted data).

In this study, we investigated the effect of parental (maternal and or paternal) morphine exposure-10 days before gestation-on the depression state of the offspring. Also, we evaluated chronic mild stress-induced depression in offspring by using chronic corticosterone injection as a model of mild chronic stress.

## 2. Methods

### 2.1. Study animals

Adult male and female Wistar rats (7 weeks old) were purchased from Pasteur Institute and kept in cages (four rats in each cage). All animals were maintained at controlled light-dark cycle (12:12 h, lights on at 7.00 AM). The temperature was kept at 23±1°C, and the humidity was constant. They had free access to food and water except during the behavioral test. After two weeks of familiarization, the animals were randomized into four groups. All experiments were approved by Tehran University of Medical Sciences Ethics Committee, matching the national guidelines for animal care.

### 2.2. Experimental procedure

#### 2.2.1. Morphine-exposure protocol

**Figure 1.**
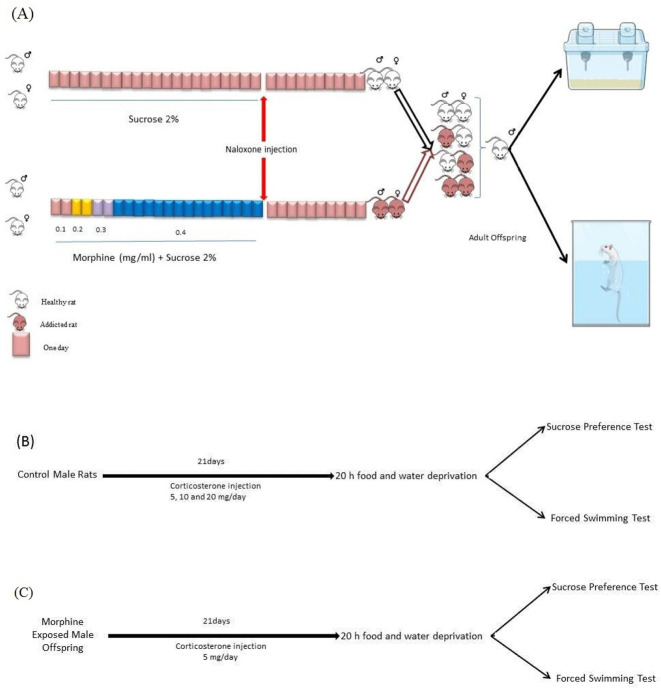
Flow chart of study design

Twenty-four male and twenty-four female Wistar rats were exposed to oral morphine sulfate (Temad, Iran) administration. Oral morphine administration protocol performed as described before (Zanjani & Sabetkasaei, 2010). Morphine sulfate was given in increasing concentrations, as mentioned in Table 1. Sucrose (2%) was added to morphine to diminish the bitter taste of morphine. The control group comprising eight male and eight female rats only received sucrose (2%). Morphine administration and mating protocol are shown in Figure 1.

**Table 1.** Morphine concentration for oral morphine administration

**Day**	**Morphine Concentration, mg/mL**
1–2	0.1
3–4	0.2
5–6	0.3
7–21	0.4

#### 2.2.2. Mating protocol

As shown in Figure 1A, ten days after the last morphine intake, the animals were prepared for mating as described below: 1. Healthy male and healthy female; 2. Healthy female and morphine-abstinent male; 3. Healthy male and morphine-abstinent female; and 4. Morphine-abstinent male and female.

In each group, one male and one female rat were put in one cage. After parturition, the numbers of live and dead births were recorded. During lactation, maternal care and weight of the infant was monitored. Male offspring grouped into four: 1. Animals with both healthy parents (control); 2. Paternal Morphine-Exposed offspring (PME); 3. Maternal Morphine-Exposed offspring (MME); and 4. Offspring with both morphine-exposed parents (PME+MME).

### 2.3. Behavioral tests

#### 2.3.1. Sucrose Preference Test

In laboratory rodents, the two-bottle choice paradigm is usually used for sucrose preference as an index of anhedonia, particularly in stress-based models of depression (Overstreet, 2012). Preference is calculated as the sucrose intake (volume of liquid consumption) divided by the total fluid intake.

All animals should be deprived of water and food 20 hours before the behavioral test. In this time, each rat is placed in the cage individually. Animals were transferred to testing room 30 minutes before starting the test. In the test day, each rat can choose between two bottles: sucrose dissolved in water (1%) and tap water. The bottles were put across left and right sides of the cages. At the end of one hour, the volume of sucrose and water consumption was measured for each rat, and sucrose preference was calculated as follows: the sucrose preference (%)=[sucrose consumption/ (sucrose consumption+water consumption)] *100.

#### 2.3.2. Forced Swimming Test

Forced Swimming Test (FST) is a behavioral test to assess depression-like behavior in rodents (Porsolt, Le Pichon, & Jalfre, 1977). Briefly, a rat is placed in a Plexiglas cylinder (60 cm height, 30 cm diameter) filled with water with a height of 30 cm. Water temperature is adjusted to 25° C. Each rat is allowed to swim for 5 minutes, and a video camera took the recording from above the cylinder. The latency time to immobility and total time of immobility (lack of motion of the whole body) are used as an index of depression-like behavior.

#### 2.3.3. Inducing mild chronic stress

We used repetitive corticosterone injection as a chronic stressor. All animals were handled once a day for seven days before corticosterone injection. As shown in Figure 1B and 1C, the rats were treated with corticosterone daily for 21 days. All behavioral tests were performed 24 h after the last injection.

Corticosterone (Sigma-Aldrich, UK) was dissolved in 0.9% saline with 2% (x)-Sorbian mono-9-octadecenoate poly(Oxy-1, 2-ethanediol) (Tween-80; Sigma-Aldrich) and injected subcutaneously once per day between 9:00 AM and 11:00 AM. All animals were weighed every day. Corticosterone was injected in 5, 10, and 20 mg/kg dose to drug-naive rats (Figure 1B) and the non-effective dose was chosen for offspring with morphine-exposed parent(s) (Figure 1C).

### 2.4. Statistical analysis

We used SPSS version 21 for data analyses. The collected data were analyzed with 1-way ANOVA. Tukey’s multiple comparison test was used for finding differences between groups. The obtained data were expressed as Mean±SD of eight animals.

## 3. Results

The mortality rate increase in the offspring of the morphine-abstinent parent(s): As shown in Table 2, mortality rate increased among the offspring of morphine-exposed parent(s) (χ^2^=31.889, P <0.001). There were no differences in weight or number of infants per parturition among groups. Depression-like behavior increase in offspring of morphine-abstinent rats:

**Table 2.** Parental morphine administration before gestation increase mortality rate in offspring

**Group**	**Mortality Rate (%)**
Control	1.1
PME	12.5
MME	25.5
PME+MME	29

**Figure 2.**
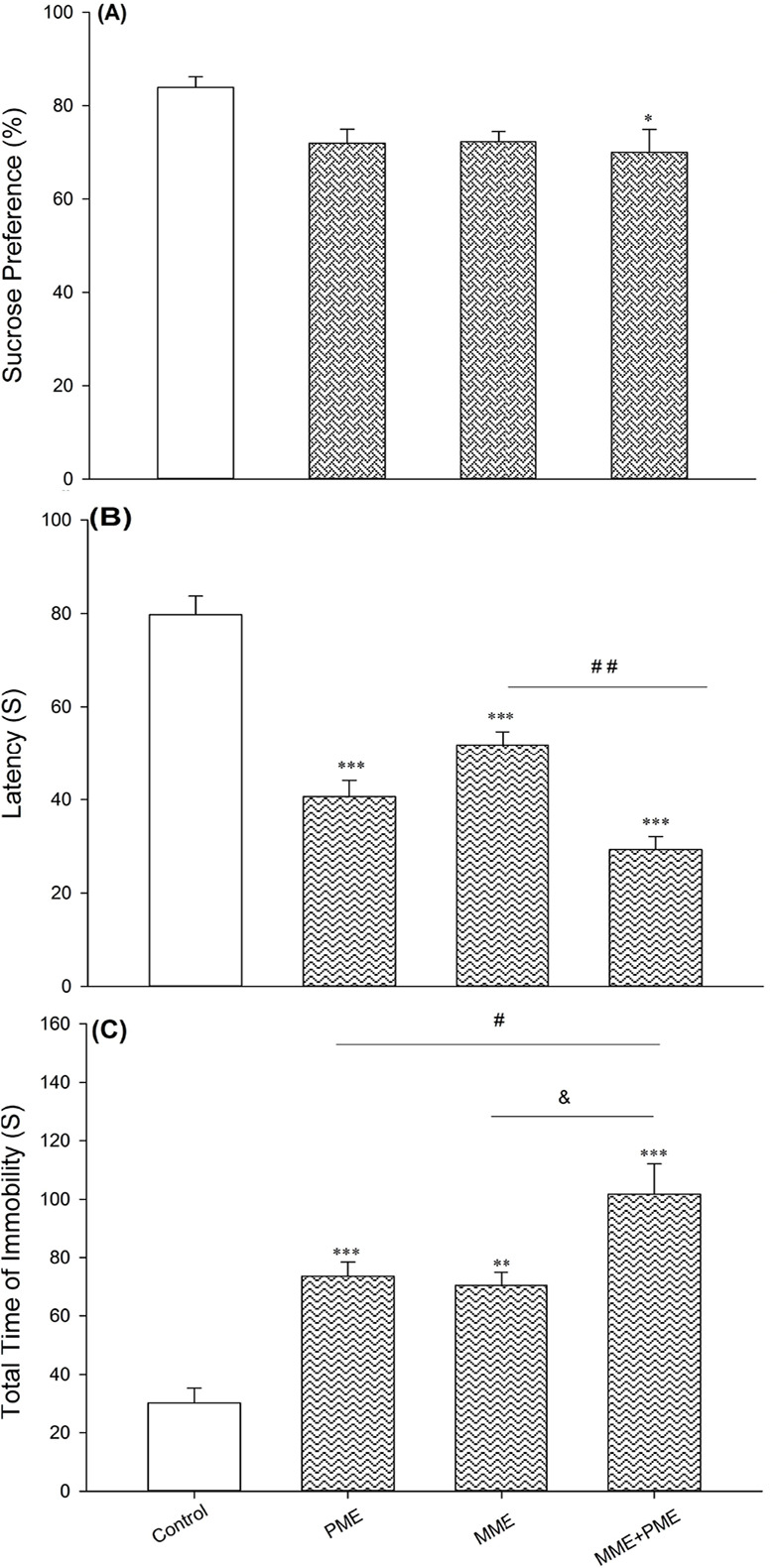
Sucrose Preference Test A. The latency to immobility; B. The total time of immobility; C. Morphine-abstinence-derived offspring. PME; Paternal morphine exposure, MME; Maternal morphine exposure. Values are presented as Mean±SD for eight rats in the control, MME, PME, and MME+PME groups. ^*^ P<0.05; ^**^ P<0.01; ^***^ P<0.001

Figure 2 shows depression-like behavior in the offspring of paternal and or maternal morphine-exposed parent(s) compared with the offspring of drug-naive parents. As shown in Figure 2A, the offspring of morphine-abstinent parents have low sucrose preference compared to the offspring of the drug-naive parent(s) (F_3,28_=3.766, P=0.02). In FST, both latency to immobility and the total time of immobility changed in the offspring of one and or two morphine-abstinent parent(s) (panel C and D). Latency time decreased in offspring of one and or two morphine-abstinent parent(s) in comparison to the offspring of drug-naive animals (F_3,28_=42.833, P<0.001).

Animals with both morphine-abstinent parents showed a significant reduction in latency time compared with maternal morphine-exposed animals (P<0.01). Total time of immobility increased among the offspring of morphine-exposed parent(s) (F_3,28_=19.686, P<0.001). The total time of immobility increased in morphine-exposed parents in comparison with one morphine-exposed parent (P<0.01). Chronic corticosterone administration increased depression-like behavior dose-dependently.

**Figure 3.**
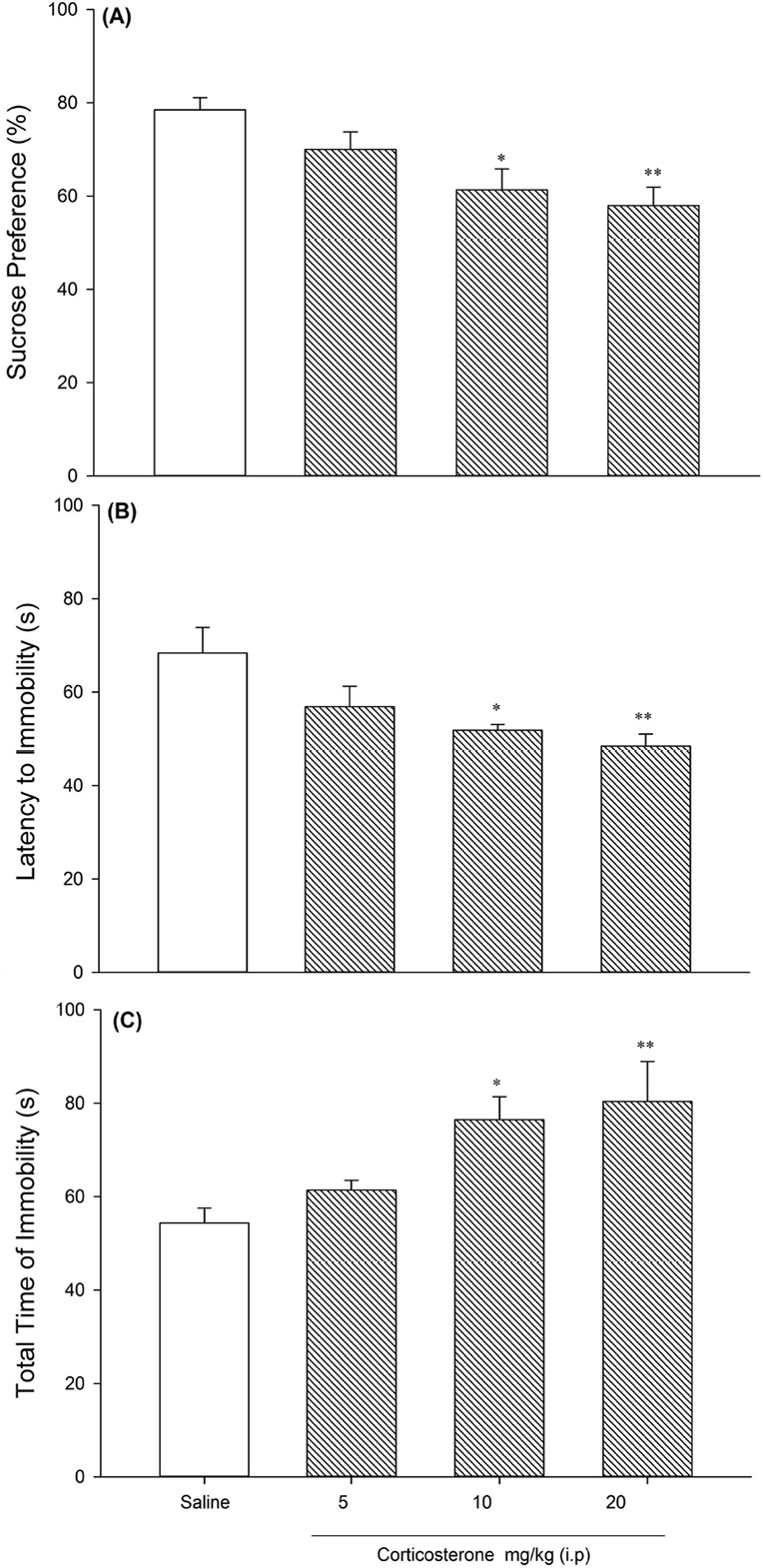
Sucrose Preference Test A. The latency to immobility; B. The total time of immobility; C. 21 days corticosterone-treated (5, 10, and 20 mg/kg) and saline-treated rats (8 for each group). Values are presented as Mean± SD. ^*^ P<0.05; ^**^ P<0.01

Figure 3 demonstrated the chronic corticosterone administration increased depression-like behavior in drug-naive animals dose-dependently. Figure 3A revealed that sucrose preference decreased in corticosterone-received rats compared with the saline-treated rats (F_3,28_=6.025, P=0.003]. The latency to immobility and total time of immobility respectively decrease and increase in chronic corticosterone administration dose-dependently in FST (F_3,28_=5.260, P=0.005; F _3,28_=5.386, P<0.005) (Figure 3B and 3C). The weight of each rat recorded daily; there were no changes in weight loss between groups (data not shown).

**Figure 4.**
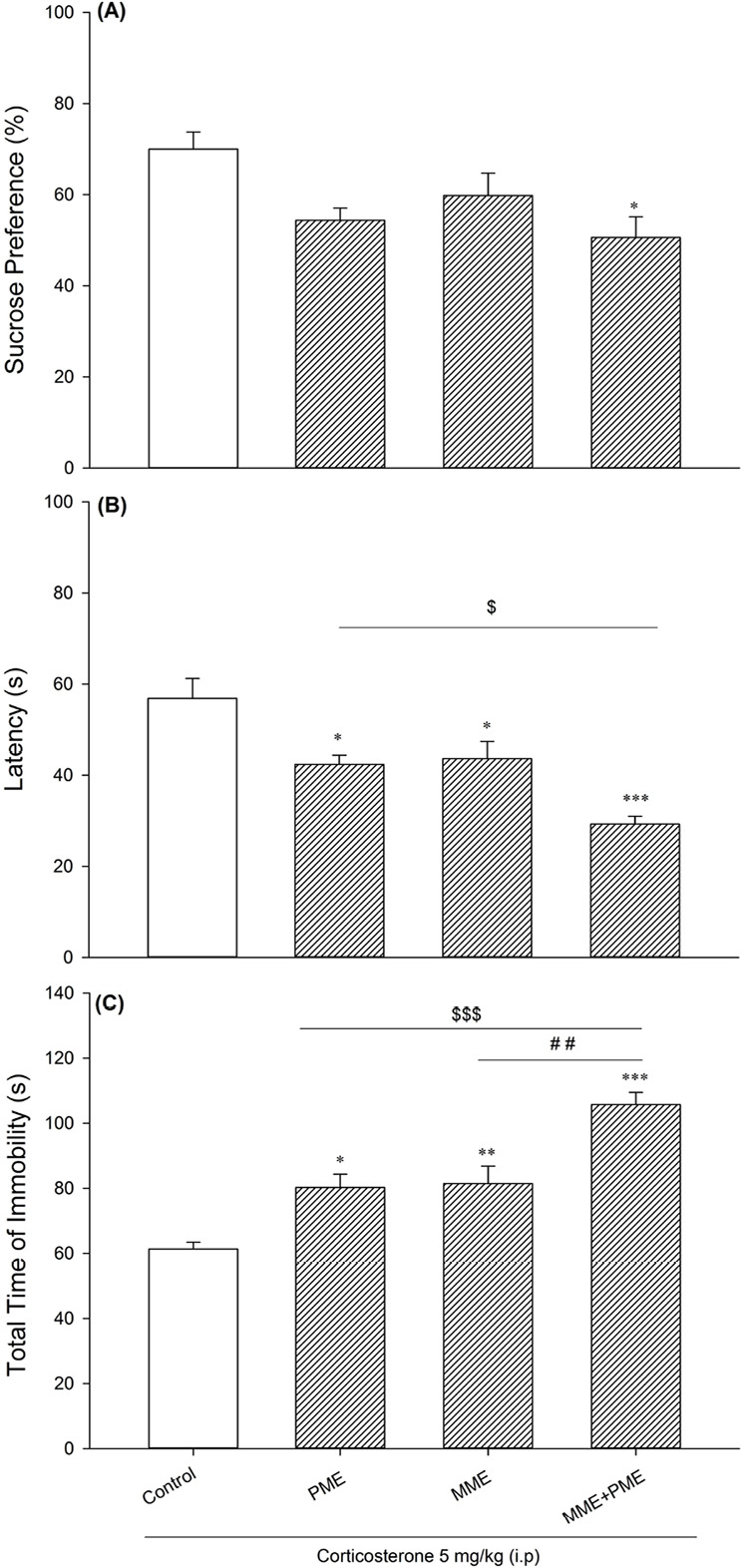
Sucrose Preference Test A. The latency to immobility; B. Total time of immobility; C. The MME, PME, MME+PME and control group which received 21 days corticosterone (5 mg/kg). Values are presented as Mean±SD for eight rats in each group. ^*^ P<0.05; ^**^ P<0.01; ^***^ P<0.001; ^$^ P<0.05; ^$$$^P<0.001 compared to the PME group; ^##^ P<0.01 compared to the MME group.

Chronic mild stressor increased depression-like behavior in the offspring of morphine-exposed rats:

Figure 4 shows that non-effective dose (5 mg/kg) of corticosterone increases depression-like behavior in the offspring of paternal and or maternal morphine-exposed animals. Figure 4A revealed that sucrose preference (in confronting with mild chronic stressor) only changed in the offspring of both morphine-abstinent parents compared with offspring of drug-naive rats (F_3, 28_=4.312, P=0.013). FST established that offspring of the morphine-exposed parent(s) are more susceptible to depression in opposing mild chronic stress compared to the control group (latency to immobility: F_3,28_=12.499, P<0.001; the total time of immobility: F_3,28_=20.848, P<0.001). There was no statistical difference between weight loss among groups (data not shown).

## 4. Discussion

Previous studies indicate that parental morphine exposure can disrupt neurochemical and neurophysiological features in the offspring (Cicero et al., 1991; Sarkaki, Assaei, Motamedi, Badavi, & Pajouhi, 2008). Also, our previous work reveals that parental morphine exposure before gestation leads to memory deficit, changing in pain perception, and increasing tolerance to morphine in male offspring (data not published yet). In this study, we found that parental morphine exposure could impress depression in offspring. The accumulating data indicate that μ-opioid receptor and β-endorphin are expressed in the male reproductive organ (Albrizio, Guaricci, Calamita, Zarrilli, & Minoia, 2006). Furthermore, μ-, κ- and δ-opioid receptors are expressed in the oocyte (Agirregoitia et al., 2012), so morphine can induce the trans-generational effect.

As mentioned previously, a variety of psychopathologies like depression, substance abuse, and suicidal attacks are common among children with addicted parents (Balsa et al., 2009; Christoffersen & Soothill, 2003). According to Fuller-Thomson study, children with alcoholic or addicted parents, experience depression 69% more than other children (Fuller-Thomson, Katz, Phan, Liddycoat, & Brennenstuhl, 2013) these studies have not examined if gender differences exist nor have they controlled for a range of potential explanatory factors. Using a regionally representative sample of 6268 adults from the 2005 Canadian Community Health Survey (response rate=83%. Additional investigation on laboratory animals has verified that morphine exposure before gestation alters the endogenous opioid system in the offspring (Cicero et al., 1991).

Paternal morphine exposure leads to increasing β-endorphin in hypothalamus and corticosterone level in serum in the female offspring (Cicero et al., 1991). Moreover, maternal morphine exposure can attenuate quinpirole-induced corticosterone in both the first and second generation male offspring (Byrnes, Johnson, Carini, & Byrnes, 2013). As approved in human studies (Fuller-Thomson et al., 2013), we confirmed that the depression rate was higher among offspring with morphine-exposed parent(s) in comparison to offspring of the control parents. Additionally, low sucrose preference in the offspring of both morphine-exposed parents, decrease in latency to immobility and an increase in the total time of immobility in FST prove our hypothesis.

Johnson et al. reported that repeated corticosterone injections increased depression-like behavior dose-dependently, and they suggested that repeated corticosterone injections dysregulated Hypothalamic-Pituitary-Adrenal (HPA) axis and consequently produced depressive disorder (Johnson, Carini, Schenk, Stewart, & Byrnes, 2006). We also found that the offspring of morphine-exposed rats were more vulnerable to depression in response to mild chronic stressor like low doses of corticosterone. The obtained data indicate that administration of corticosterone with low dose (5 mg/kg) does not induce depression-like behavior in the offspring of the healthy rats but can cause depression-like behavior in the offspring of the morphine-exposed rats.

This effect may have occurred following the HPA axis dysregulation (Cicero et al., 1991). Mild stressor might increase corticosterone level in the offspring of morphine-exposed rats more than offspring of the healthy rats, and probably such effect is mediated via the enhanced level of corticosterone in plasma. A study by Houshyar and colleagues reveals that chronic morphine administration results in the persistent elevation of basal plasma corticosterone level approximately ten times more than the control group (Houshyar, Galigniana, Pratt, & Woods, 2001).

Hippocampus is an important area involved in depression (Sheline, Mittler, & Mintun, 2002). Sarkaki et al. reported that paternal morphine exposure disrupted hippocampal synaptic plasticity in male offspring (Sarkaki et al., 2008). The Paraventricular Nucleus (PVN) of the hypothalamus contains neurons with corticotropin-releasing factor, and these neurons integrate information pertinent to stress. In PVN, excitatory afferent from the amygdala and inhibitory afferent from hippocampus are prominent. Moreover, ascending monoamine inputs, inputs from the periphery, and inhibitory inputs from circulating glucocorticoids are convergent in PVN. Chronic stress and continuous rise of glucocorticoids decrease expression of Brain-Derived Neurotrophic Factor (BDNF) in the hippocampus.

BDNF has a significant role in neurons survival, and its decreasing may contribute to CA3 atrophy. Losing CA3 neurons increased susceptibility to psychiatric diseases such as depression and anxiety (Nestler, Hyman, & Malenka, 2015). Likewise, prenatal morphine exposure decreases Long-Term Depression (LTD) in CA1 pyramidal neurons (Yang et al., 2003). These changes in neuronal plasticity in the hippocampus may affect depression-like behavior in the offspring of the morphine-exposed parent(s). Evidence proved that morphine exposure before parturition or during mothering change maternal care (Bridges & Grimm, 1982; Miranda-Paiva, Nasello, Yin, & Felicio, 2001; Slamberova, Szilagyi, & Vathy, 2001). Other investigations revealed that morphine administration in adolescence could alter maternal-care-like nursing and contact time during lactation (Johnson, Carini, Schenk, Stewart, & Byrnes, 2011).

Finally, in addition to the role of HPA axis, psychological changes in the offspring of addict rats, another possible reason for such depressive disorder is an epigenetic transmission. Morphine can directly affect epigenetic germ cells. These changes impress developmental processes in the embryo. The mechanisms which cause such trans-generational effect are unknown, and other investigation should be conducted to find exact mechanisms of such effects.
